# Lectures on the Theory and Practice of Surgery

**Published:** 1845-08

**Authors:** 


*Lectures on the Theory and Practice of Surgery.* By the late Abraham Colles, M. D., for thirty-four years Professor of Surgery in the Royal College of Surgeons in Ireland. Edited by Simon McCoy, Esq., F. R. C. S. I. 8vo., pp. 420 ; Philadelphia: Ed. Barrington and George D. Haswell, 1845.

This is another valuable reprint, issued by the publishers of the Select Medical Library, and for which we are indebted to the just discrimination of its able editor, Dr. John Bell.

For many years past, the Medical School of Dublin has occupied a conspicuous position in the estimation of physicians, every where. No other city in the world, probably, can boast of a greater number of able and learned members of the medical profession, in proportion to the population, than the Irish Metropolis. To have been a distinguished lecturer in such a school, and long regarded as at the head of the profession where the members are so accomplished, is a sufficient guarantee for the value of the lectures he delivered. The present publication, we are told, has been compiled from the notes of his lectures, taken by one of his class, during his attendance on several courses, and carefully collated with the manuscripts of others of his pupils, so that, although it has never received the correction or sanction of Dr. Colles, it may be presumed to be a true exposition of his opinions and practice. Indeed, the European publishers allege that it contains "a true and correct record, not only of the matter of the invaluable lectures delivered by Mr. Colles, but of the manner of the lecturer." Being notes of lectures, the style of the work is colloquial, but without being unnecessarily diffuse. Little is said about operations, at least as to details; and yet, a glance will show that, throughout, it is mainly practical—an exposition of the personal experience of the lecturer.

The doctrines inculcated in these lectures are, in the main, such as receive the approbation of the distinguished Surgeons of the present time. On some points, however, they are behind the day, not merely in theory but in practice. We have an instance of this in his advocacy of mercury, constitutionally administered, in the treatment of primary venereal sores. It is not our purpose, however, to discuss the moot points contained in the work, but to express our high appreciation of its general merits. No Surgeon who aims at distinction in his profession, will fail to make himself acquainted with the opinions and experience of Colles.

